# Canaloplasty in Corticosteroid-Induced Glaucoma. Preliminary Results

**DOI:** 10.3390/jcm7020031

**Published:** 2018-02-11

**Authors:** Paolo Brusini, Claudia Tosoni, Marco Zeppieri

**Affiliations:** 1 Department of Ophthalmology, “Città di Udine” Health Center, Viale Venezia, 410, 33100 Udine, Italy; c.tosoni@libero.it; 2 Department of Ophthalmology, “Azienda Ospedaliero-Universitaria” “Santa Maria della Misericordia” Hospital of Udine, P.le S. Maria della Misericordia, 15, 33100 Udine, Italy; markzeppieri@hotmail.com

**Keywords:** canaloplasty, non-perforating surgical procedures, corticosteroid-induced glaucoma, Schlemm’s canal

## Abstract

Purpose: to present the mid-term results of canaloplasty in a small cohort of corticosteroid glaucoma patients. Material and Methods: Nine eyes from seven patients with various types of corticosteroid glaucoma in maximum medical therapy underwent canaloplasty. Patients underwent complete ophthalmic examination every six months. Success was defined as: post-operative intraocular pressure (IOP) ≤ 21 mmHg and ≤ 16 mmHg without (“complete success”), and with/without medical treatment (“qualified success”). The IOP reduction had to be ≥ 20. The number of medications before and after surgery was considered. The follow-up mean period was 32.7 ± 20.8 months (range 14–72 months). Results: The pre-operative mean IOP was 30.7 ± 7.2 mmHg (range: 24–45). The mean IOP at 6 and 12-month follow-up was 13.1 ± 2.6 mmHg, and 13.7 ± 1.9 mmHg, respectively. Qualified and complete success at 6 and 12 months was 100% for both of the two definitions. The number of medications used preoperatively and at the 12-month follow-up was 4.3 ± 0.7, and 0.2 ± 1.0, respectively. No serious complication was observed. Conclusions: The mid-term results of canaloplasty in patients with corticosteroid-induced glaucoma appear to be very promising. Canaloplasty should be considered as a possible alternative to filtering surgery in this form of glaucoma, when medical therapy is not sufficient to maintain the IOP within reasonable limits.

## 1. Introduction

Corticosteroid-induced glaucoma is a quite common form of secondary glaucoma due to either systemic or, more frequently, topical, peri- or intraocular administration of glucocorticoids in predisposed subjects [1,2,3,4,5]. It is known that corticosteroids raise intraocular pressure (IOP) by lowering the facility of aqueous outflow. Quite a high percentage of normal subjects (ranging from 5% to over 40% depending on the definition of corticosteroid-responders [6,7] may undergo a significant increase of IOP after using topical corticosteroids for several days. The increasing use of intravitreal injections of triamcinolone acetonide and intravitreal implants of dexamethasone for exudative maculopathies will probably exacerbate this problem. A secondary glaucoma can develop in some cases, even though for most patients the IOP returns to baseline after ceasing steroid use. If traditional medical therapy is not able to lower IOP within the safe range, structural and functional damage can quickly develop. In these cases, a laser trabeculoplasty can be attended [8,9,10], but more often a surgical treatment must be performed before serious visual impairment occurs. Trabeculectomy with intra-operative antimetabolites is still considered to be the gold standard surgical procedure for different types of glaucoma, including corticosteroid-induced glaucoma [11,12]. This technique is simple to perform and effective, however, several early and late potentially serious complications can occur. In particular, problems related to the subconjunctival bleb, and the frequent development of a cataract can be particularly disturbing in young patients, who are often the subjects that develop corticosteroid-induced glaucoma.

Canaloplasty is a non-perforating bleb-less technique, introduced some years ago, in which a 10-0 prolene suture is positioned and tensioned within Schlemm’s canal, previously dilated with a viscoelastic agent, thus facilitating aqueous outflow through natural pathways [13,14].

The purpose of this study is to present the mid-term results of canaloplasty in a small cohort of patients with corticosteroid-induced glaucoma resistant to medical management.

## 2. Experimental Section

In this non-randomized prospective study, 9 eyes from 7 patients with uncontrolled corticosteroid-induced glaucoma under maximum tolerated medical therapy underwent canaloplasty under peribulbar anesthesia. Surgery was performed either at the Department of Ophthalmology at the Azienda Ospedaliero-Universitaria “Santa Maria della Misericordia” Hospital in Udine (Italy), or at the “Città di Udine” Health Center in Udine (Italy) by one surgeon (PB), with a 10-year experience with this type of surgery, from February 2008 to July 2016.

All patients provided written informed consent. The protocol was approved by the institutional review board or ethics committee.

Inclusion criteria included: (1) patients with ocular hypertension due to corticosteroid use; (2) IOP ≥ 24 mmHg with maximum tolerated medical therapy after stopping corticosteroid use (in the case of topical administration); (3) open irido-corneal angle; (4) age over 18 years. Patients with or without typical optic nerve alterations, and with or without glaucomatous visual field defects were considered.

Exclusion criteria included: (1) elapsed time after steroids stopping shorter than 3 months; (2) age under 18 years; (3) other possible causes of glaucoma (i.e., pseudoesfoliation, previous trauma, etc.); (4) previous ocular surgery, apart from cataract and intravitreal injections; (5) refusal to undergo surgery.

The patients’ demographic data, causes of secondary glaucoma, preoperative IOP, number of drugs used, visual functions and length of the follow-up at the last visit and other clinical data are reported in Table 1.

The canaloplasty surgical technique is well known and has been extensively reported in the literature [15,16,17,18,19]. Briefly, surgery starts with a fornix-based conjunctival flap, and a 3 × 4 mm superficial scleral flap, which is dissected forward into the clear cornea for 1.5 mm. A deep scleral flap is then created, in order to open the Schlemm’s canal. The deep scleral flap is removed and the two openings of the canal are dilated with hyaluronic acid in order to cannulate the Schlemm’s canal, by means of a special microcatheter (iTrack by iScience Interventional, Menlo Park, CA, USA), connected to a flickering red laser light source for easy identification of the distal tip through the sclera. The microcatheter is inserted and pushed forward within Schlemm’s canal for the entire 360° until it comes out of the other end of the canal opening. A 10-0, prolene suture is then tied to the distal tip and the microcatheter is withdrawn back through the canal in the opposite direction. During this maneuver, a small amount of high-molecular weight viscoelastic agent (Healon GV, Abbot Medical Optics, Santa Ana, CA, USA) is delivered within the canal every two hours of circumference, using a special screw-driven syringe. The suture is then knotted under tension in order to inwardly distend the trabecular meshwork. The superficial scleral flap is sutured with seven 10-0 Polyglactin 910 stitches to ensure a watertight closure in order to prevent any bleb formation. The conjunctival flap is then sutured with two 10-0 sutures to complete the surgery.

All patients underwent a visit once a week for the first month in order to measure IOP and detect any possible postoperative complication, then went on to have a complete ophthalmic examination every six months, including slit-lamp examination, best corrected visual acuity (BCVA), IOP measurement with Goldmann applanation tonometer, fundus examination with a 78 D Volk lens, visual field testing (Humphrey Field Analyzer (Carl Zeiss Meditec Inc. Dublin, CA, USA) 30-2 SITA standard test), retinal nerve fiber layer assessment with spectral-domain OCT and gonioscopy. Moreover, the corneal astigmatism was measured after one week and one month by means of a keratometer of Javal and corneal topography. Visual field damage severity was assessed using the Glaucoma Staging System 2 (P. Brusini, Italy) [20]. The definition of success was based on two different criteria: post-operative IOP ≤ 21 mmHg and ≤ 16 mmHg. When this goal was obtained without any medical treatment, the success was defined as “complete”. When the same IOP levels were obtained with or without medical treatment, the success was defined as “qualified”. Moreover, the IOP reduction had to be ≥20% for defining a case as successful. The number of medications before and after canaloplasty was also taken into consideration.

Differences between test results were calculated using the paired *t*-test for variables that showed a normal distribution. The statistical analysis was performed using SPSS 11.0 (IBM Analytics, Chicago, IL, USA). Statistical significance was defined as *p* < 0.05.

## 3. Results

**Figure 1:**
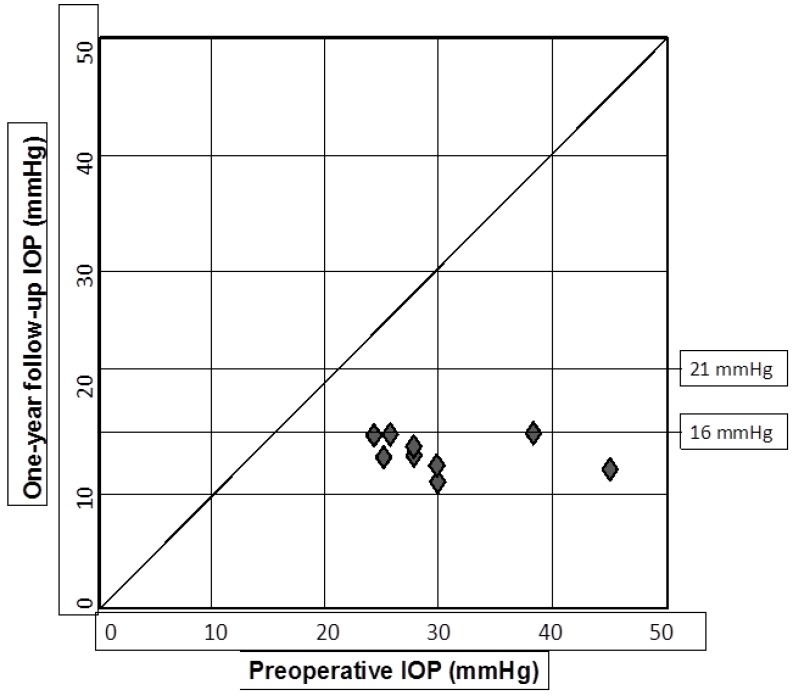
Pre- and one-year post-operative IOP values.

The entire standard procedure could be performed as planned in all of the nine eyes. Follow-up ranged from 14 to 72 months (mean: 32.7 ± 20.8). The mean pre-operative IOP was 30.4 ± 6.8 mmHg, ranging from 24 to 45 mmHg. The mean IOP after 6 and 12 months was 13.1 ± 2.6 mmHg, and 13.7 ± 1.9 mmHg, respectively, ranging 11 to 18 mmHg (paired *t*-test, *p* = 0.0001). The mean IOP reduction from baseline after 6 and 12 months was of 56.9% and 54.9%, respectively. The IOP values at various follow-up sessions within a period of 36 months are shown in Table 2. The scatter plot in Figure 1 shows the pre- and one-year post-operative IOP values. After the 6 and 12-month follow-up, a complete and qualified success, was obtained in all 9 eyes, using both the definitions of success (IOP ≤ 21 mmHg and ≤ 16 mmHg), with an IOP within normal limits during the entire follow-up period, except for one eye that showed an increase of IOP after two years, successfully controlled with medical therapy. The number of medications used pre- and at the 12-month follow-up was 4.3 ± 0.7, and 0.2 ± 1.0, respectively (difference statistically significant, *p* < 0.001). Only one patient (11%) was under IOP-lowering drops after one year, but medical treatment was needed in both the two patients which reached a five-year follow-up. No patient required adjunctive surgical procedures. Gonioscopy showed that the prolene suture remained correctly positioned within the Schlemm’s canal for the entire follow-up period in all cases. At the last visit, visual acuity worsened of two lines in two eyes (case #1 and case #2), depending on retina conditions, and improved of three lines in one eye (case #7) due to the effects of corticosteroid treatment. A reliable visual field testing was not possible in three eyes due either to a poor visual acuity (case #9) or to artifacts related to retinal disease (case #1 and #2). Before surgery, visual field was normal in one case (#5), showed only slight defects in four cases, whereas only small islands of vision were present in another case (#9). All of these defects remained stable over time. Optic nerve appearance (normal in all eyes but one) and retinal nerve fiber layer did not show any significant change during the follow-up period.

The early post-operative complications (within four weeks from surgery) included: microhyphema in two eyes (22.2%); hypotonus (IOP < 5 mm/Hg) in one eye (11.1%); and, IOP spikes > 10 mmHg in one case (11.1%). A transient decrease in visual acuity in the first weeks after canaloplasty was a rather common finding, which was due to an induced according to-the-rule astigmatism which can reach five diopters, but usually disappear within one month. No surgery-related complications were observed after two months.

## 4. Discussion

Surgery is sometimes needed to control ocular hypertension and delay damage progression in patients with corticosteroid-induced glaucoma, especially considering that visual field defect progression can be fast and severe if IOP is very high. However, unlike from patients with primary open-angle glaucoma or pseudoesfoliation glaucoma, which often show advanced visual field loss, patients with corticosteroid-induced glaucoma usually have normal optic nerves and visual fields at the beginning. For this reason, an IOP in the mid-teens is usually adequate in order to avoid any structural and/or functional damage. In this type of patient, even with very high pre-operative IOP levels, non-filtering surgical procedures, such as goniotomy [21], trabeculotomy [22,23,24,25], trabecular stents [26], viscocanalostomy [27] or deep sclerectomy [28] may be an interesting option, even if they are less effective than trabeculectomy in lowering IOP, considering the lower risk of complications. Nowadays, canaloplasty should be considered as a step ahead of these procedures with very interesting long-term outcomes in various forms of open-angle glaucoma [15,16,17,18,19].

Our mid-term results in a small cohort of patients with corticosteroid-induced glaucoma unresponsive to medical therapy appear to be particularly good in comparison with other types of glaucoma, where the mean IOP usually ranges between 15 and 17 mmHg, with a percentage of success after one year ranging between 60% and 95%, depending on the definition of success used [14,15,16,17,18,19]. In particular, if a cut-off of ≤ 16 mmHg is taken in order to define successful cases, the percentage of qualified success reported in literature is about 50% in comparison with the 100% obtained in our nine cases.

The reasons for this favorable behavior are probably various and include: (1) histopatologic studies in corticosteroid-induced glaucoma demonstrated an increased density of the cribriform meshwork and thinning of the endothelial lining of Schlemm’s canal [29,30]; in cases of elevated IOP, a collapse of aqueous plexus and collector channel ostia obstructed by herniation was observed in bovine eyes [31], resulting in a decrease in the effective filtration area. Canaloplasty is able to overcome this obstacle, allowing the restoration of the aqueous humor outflow; (2) patients with this type of glaucoma are usually relatively young with well-functioning aqueous humor pathways, which is a fundamental requirement to obtain satisfactory results after canaloplasty; (3) all of our patients were under medical therapy for a short period before the operation; it is well known that topical therapy for glaucoma has negative effects in all glaucoma surgeries.

It should be noted, however, that the percentage of patients which require a pharmacological therapy, even at a lower dosage, to maintain adequate IOP control seems to increase with time.

Even if the results we obtained are very promising, it should be remembered that this was a non-randomized study with a small sample of patients without a control group. Another limitation of our study is that both eyes of two patients have been considered. Even if this could be incorrect from a statistical point of view, considering the small number of patients treated, we decided to describe all cases we treated with this surgical procedure. The study is currently still underway. New patients with corticosteroid-induced glaucoma that fit the inclusion criteria are being added and follow-up data of existing patients are being constantly updated to provide long term results and a larger cohort for our future study. Regarding these patients, multicentric randomized studies with a larger population, where canaloplasty is compared to gold standard surgery (trabeculectomy), are needed to draw more definite and robust conclusions.

## 5. Conclusions

Canaloplasty is a very promising surgical technique in eyes with high IOP, which is usually the case in patients with corticosteroid-induced glaucoma. In our small cohort of patients, postoperative IOP was able to be maintained within physiological values, even if some medical therapy is occasionally still required. Considering that ocular hypertension is the main risk factor for structural and functional damage in corticosteroid-induced glaucoma, target IOP may not need to be very low to avoid the onset or the progression of the damage. Even if the sample taken into consideration in our study was limited, the good outcomes and the low rate of complications observed with this non-perforating procedure are very encouraging and could entice glaucoma specialists to consider early surgical treatment in the management of this kind of patient.

## Figures and Tables

**Table 1:** Patient data before surgery.

Case	Sex	Age	Cause of OHT		Pre-Op IOP	No. of Meds	VA	VF Stage (GSS 2)	Follow-Up Length (Months)
1	M	49	Dex IV	CRVO	38	4	6/10	NA	31
2	M	45	Triam IV	EM	30	6	3/10	NA	16
3	M	48	Dex drops	AC	26	4	8/10	1	72
4	M	48	Dex drops	AC	24	4	9/10	1	60
5	F	18	Dex drops	AC	28	4	10/10	1	18
6	F	18	Dex drops	AC	30	4	10/10	1	23
7	M	69	Dex IV	CRVO	28	4	5/10	5	14
8	F	69	Dex IV	EM	45	5	2/0	0	19
9	M	34	Dex drops	PK	25	4	CF	NA	41
Mean (±SD)		44.2 ± 10.6			30.4 ± 6.8	4.3 ± 0.7			32.7 ± 20.9

OHT = ocular hypertension; Pre-op IOP = preoperative intraocular pressure; Dex IV CRVO = Intravitreal dexametason for central retinal vein occlusion; Triam IV EM = Triamcinolone acetonide for exudative maculopathy; Dex drops AC = dexametason drops for allergic conjunctivitis; Dex drops PK = dexametason drops after perforating keratoplasty; VA = visual acuity; VF = visual field; GSS 2 = Glaucoma Staging System 2; NA = not applicable; CF = counting fingers; SD = standard deviation.

**Table 2:** Mean post-operative intraocular pressure (IOP).

Time Point	IOP, Mean ± SD (mmHg)	No. of Eyes
Preoperative	30.4 ± 6.8	9
1 month	12.6 ± 1.9 *	9
6 month	13.2 ± 2.6 *	9
12 month	13.7 ± 1.9 *	9
24 month	16.8 ± 6.3 *	5
30 month	15.7 ± 2.5 *	4
36 month	15.7 ± 2.3 *	3
42 month	15.5 ± 2.1 *	2
48 month	17.0 ± 4.2 *	2
54 month	16.0 ± 1.4 *	2
60 month	14.5 ± 0.7 *	2

* *p* < 0.0001 vs. preoperative values.
